# Predictive Value of N-Terminal Pro B-Type Natriuretic Peptide for Short-Term Outcome of Cardioversion in Patients with First-Diagnosed or Paroxysmal Atrial Fibrillation Presenting to the Emergency Department

**DOI:** 10.3390/biomedicines12122895

**Published:** 2024-12-19

**Authors:** Antonios Diakantonis, Christos Verras, Sofia Bezati, Vasiliki Bistola, Ioannis Ventoulis, Maria Velliou, Antonios Boultadakis, Ignatios Ikonomidis, John T. Parissis, Effie Polyzogopoulou

**Affiliations:** 1 University Department of Emergency Medicine, Attikon University Hospital, National and Kapodistrian University of Athens, 12462 Athens, Greece; christos.verras@gmail.com (C.V.); sofiabezati@gmail.com (S.B.); vasobistola@yahoo.com (V.B.); maravelliou84@yahoo.gr (M.V.); boult_doc@yahoo.gr (A.B.); jparissis@yahoo.com (J.T.P.); effiepol@med.uoa.gr (E.P.); 2 Second Department of Cardiology, Attikon University Hospital, National and Kapodistrian University of Athens, 12462 Athens, Greece; ignoik@gmail.com; 3 Department of Occupational Therapy, University of Western Macedonia, 50200 Ptolemaida, Greece; iventoulis@uowm.gr

**Keywords:** NT-proBNP, atrial fibrillation, cardioversion, emergency department

## Abstract

**Background**: Atrial fibrillation (AF) is a common arrhythmia in the emergency department (ED). We investigated the role of N-terminal pro b-type natriuretic peptide (NT-proBNP) in predicting both the outcome of AF cardioversion and the risk of AF recurrence or persistence on the 8th (D8) and 30th (D30) day post-cardioversion. **Methods**: This prospective, observational study evaluated patients with recent-onset AF, managed by either pharmacological (PC) or electrical cardioversion (EC) in the ED. Patients were treated either immediately or electively after 3 weeks of anticoagulation. NT-proBNP assessments were performed prior to cardioversion. **Results:** Of the 148 patients enrolled, 56% had paroxysmal AF, 85% underwent immediate cardioversion and 72% received EC. Successful cardioversion to sinus rhythm (SR) was achieved in 85% of patients. Patients with successful cardioversion and those who remained free from AF on D8 had lower NT-proBNP levels compared to patients with failed cardioversion or with AF recurrence or persistence on D8 [day of cardioversion, D0: SR vs. non-SR, 387 (127–1095) pg/mL vs. 1262 (595–2295), *p* = 0.004; D8: SR vs. non-SR, 370 (127–1095) vs. 1366 (718–2295), *p* = 0.002]. In multivariate analysis, higher logNT-proBNP was associated with higher risk of cardioversion failure [OR, 95%CI: 4.80 (1.58–14.55), *p* = 0.006] and AF recurrence or persistence on D8 [OR, 95%CI: 3.65 (1.06–12.59), *p* = 0.041]. ROC analysis confirmed the predictive ability of NT-proBNP for both outcomes (D0: AUC 0.735, *p* < 0.001; D8: AUC 0.761, *p* < 0.001). A cut-off value of NT-proBNP > 580 pg/mL was able to predict failure of AF conversion and occurrence of recurrent/persistent AF at D8. **Conclusions**: NT-proBNP is a promising biomarker for identifying patients presenting to the ED with recent-onset AF who run a greater risk of cardioversion failure and post-discharge AF recurrence/persistence in the immediate and short term.

## 1. Introduction

Atrial fibrillation (AF) is the most common arrhythmia in clinical practice, affecting millions of people globally [1]. It often results in emergency department (ED) visits and is associated with considerable morbidity and mortality due to thromboembolic events and related complications [2]. AF is classified as first-diagnosed, paroxysmal, persistent, or permanent and the appropriate management includes rhythm control, rate control, and anticoagulation for the prevention of thromboembolic events [3]. Rhythm control aims to restore normal sinus rhythm (SR) through pharmacological (PC) or electrical cardioversion (EC), while rate control aims to limit a rapid ventricular response rate with beta-blockers, non-dihydropyridine calcium-channel blockers, or digoxin [4]. Given the likelihood of spontaneous cardioversion of recent-onset AF to SR [5], it is plausible to estimate its probability before determining the appropriate treatment plan, through the estimation of proposed risk scores [6]. This is may be of great value in patients with special characteristics such as short duration of AF, limited recurrent episodes, normal dimensions of the atrium and without history of structural heart disease [5].

Several drugs are available for treating recent-onset AF in the ED [2,3,7,8]. Flecainide, a class IC antiarrhythmic agent, has an AF conversion rate of approximately 70% within 24 h [9]. Its availability in both oral and intravenous forms offers versatility in emergencies [10]. However, it is contraindicated in patients with structural heart disease or coronary artery disease. On the other hand, EC may be safer and even more effective than pharmacological cardioversion when applied by skilled physicians in the ED and is the recommended rhythm-control strategy in patients with hemodynamic instability [11,12,13]. However, recurrence of AF despite electrical cardioversion and/or antiarrhythmic treatment is frequently encountered [14]. Identifying patients at increased risk for failure of AF cardioversion and for recurrence of AF (or persistence of AF in case of failure of index cardioversion) through cardiac biomarkers easily assessed at the ED, would allow the implementation of appropriate follow up strategies post-ED management.

Furthermore, the role of various biomarkers, including elevated N-terminal pro-B-type natriuretic peptide (NT-proBNP), has been studied within the context of AF [15,16]. NT-proBNP, mainly studied in patients with heart failure (HF), is released in response to increased end-diastolic wall stress due to volume expansion or pressure overload [17].

Silvet et al. were the first to point out that NT-proBNP levels were elevated in patients with chronic AF, even in patients without left ventricular dysfunction [18]. This observation was corroborated in subsequent large randomized trials, which also attributed a prognostic role to NT-proBNP in terms of morbidity, mortality [19] and disease progression [20].

In parallel, various biomarkers involved in the genesis and perpetuation of AF have been described in the literature and may aid in the clarification of the underlying mechanisms of AF [21]. Likewise, C-reactive protein (CRP), interleukin-6 (IL-6) [22], and growth differentiation factor-15 (GDF-15) [23], unspecific markers of inflammation and oxidative stress, have been associated with a dismal prognosis for patients with AF. Elevation of cardiac troponins may suggest myocardial stress and necrosis due to high ventricular rates and altered contractility [24] and its measurement along with NT-proBNP has been incorporated in the risk stratification ABC-stroke score [25] so as to detect patients with increased risk of thromboembolic events and cardiovascular death. Moreover, biomarkers of fibrosis, such as galectin-3 and soluble suppression of tumorigenicity 2 protein, (sST-2), indicative of atrial remodeling, have also been associated with AF recurrence [26]. Even so, further research is needed in order to attribute the specific clinical implications of each biomarker and to establish a broad implementation of biomarker-based strategies in the management of patients with AF.

Accordingly, the aim of the present study was to investigate the role of NT-proBNP in predicting the outcome of cardioversion in the ED setting in patients without heart failure but with reduced ejection fraction (HFrEF). Furthermore, we examined its predictive value regarding early AF recurrence or AF persistence in the short term after ED management.

## 2. Materials and Methods

### 2.1. Study Population

This prospective observational single-center study was conducted at the ED of Attikon University Hospital, Athens, Greece, a tertiary university hospital that provides a full range of emergency clinical services. The study enrolled hemodynamically stable adult patients with first-diagnosed or paroxysmal AF, who were managed by PC or EC at the ED, either upon presentation to the ED (if AF duration was less than 48 h) or electively after at least 3 weeks of anticoagulation. Definitions and management of AF were based on the 2020 European Society of Cardiology (ESC) guidelines for the management of AF [3]. Exclusion criteria included age less than 18 years, previous myocardial infarction (MI) treated either with percutaneous coronary intervention (PCI) or coronary artery bypass grafting (CABG), structural heart disease, left ventricular ejection fraction (LVEF) less than 40%, hypertrophic cardiomyopathy, restrictive cardiomyopathy, or refusal to provide written informed consent. All patients or their next of kin provided written informed consent before enrollment in the study. The study was performed in accordance with the Declaration of Helsinki and was approved by the institutional review board (IRB) of the Attikon University Hospital (IRB number 283/05-05-2022).

In order to determine the sample size, post hoc power calculation was performed based on previous literature showing that recurrence of atrial fibrillation within 30 days after cardioversion is approximately 28% [27,28]. Power analysis showed that the study had a 99.1% power to detect the observed recurrence of atrial fibrillation at 30 days at an alpha level of 0.05.

### 2.2. Study Procedures

Upon presentation to the ED, the following data were obtained from each enrolled patient: demographics including age and sex; past medical history; vital signs including respiratory rate (RR), pulse oximetry blood oxygen saturation (SpO2), systolic blood pressure (SBP), diastolic blood pressure (DPB), heart rate (HR) and body temperature; and a 12-lead electrocardiogram (ECG). Venous blood was drawn from an antecubital vein prior to cardioversion and laboratory tests were performed including complete blood count, serum biochemistry [urea, creatinine, aspartate aminotransferase (AST), alanine aminotransferase (ALT), glucose, sodium (Na), potassium (K)], cardiac biomarkers including high sensitivity troponin T (hs-TnT) and NT-proBNP, thyroid-stimulating hormone (TSH), C-reactive protein (CRP), D-Dimers, international normalized ratio (INR), prothrombin time (PT), and fibrinogen. Glomerular filtration rate (eGFR) was estimated using the Chronic Kidney Disease Epidemiology Collaboration (CKD-EPI) formula [29]. All laboratory exams were performed at the hospital’s central laboratory. NT-proBNP was analyzed by means of an immunoassay method in a Preanalytics-COBAS 8000 (Roche Diagnostics, Basel, Switzerland) analyzer. Moreover, prior to study enrollment, patients underwent transthoracic echocardiography with a Venue Go 1.75–3.5 MHz scanner (GE HealthCare Ultrasound, Chicago, IL, USA) with the use of harmonic imaging. Left atrial (LA) diameter was measured in the parasternal long axis view, while LVEF was visually assessed.

At the discretion of the emergency physician, AF cardioversion strategy was either PC using intravenous (iv) flecainide, or EC by means of synchronized direct current EC. The selection of cardioversion strategy by the ED treating physician was based on age, comorbidities, possible adverse effects associated to flecainide administration and peri-anesthetic risk to the study subjects. Decision-making was also guided by patient preference. In both strategies, cardioversion was performed in the resuscitation room and under continuous monitoring of the patient’s vital signs and ECG. The iv dose of flecainide was administered according to the summary of product characteristics (SPC) of the drug, with the therapeutic regimen including administration of a loading dose of 2 mg/kg over 10–20 min up to a maximum total dose of 150 mg [30]. EC was performed after procedural sedation and analgesia (propofol 0.2–0.3 mg/kg and fentanyl 50–100 mcg/kg) with a biphasic defibrillator and antero-posterior electrode positioning, according to the European Resuscitation Council (ERC) [31] and ESC [3] guidelines for the management of patients with AF. An initial shock of 150 J was delivered and, if unsuccessful, a second and a third (if needed) attempt with 200 J was performed [32].

### 2.3. Outcomes

Patients were followed for 30 days after enrollment. The outcomes analyzed included failure of cardioversion of AF at the ED, as well as AF recurrence at 8 and 30 days after ED management in patients who were successfully cardioverted, or persistence of AF at 8 and 30 days in patients in whom index cardioversion had failed. Follow-up information from study participants was collected via follow-up visit at the investigator site. ECG was performed in order to determine rhythm status.

### 2.4. Statistical Analysis

Statistical analysis was performed using SPSS version 29.0 (SPSS, Inc., Chicago, IL, USA). Categorical variables are presented as frequencies and percentages, whereas quantitative variables are expressed as median with interquartile range (IQR). The values of laboratory variables were log-transformed for simple correlations and regression analyses, due to their highly skewed distribution. In order to examine the association between NT-proBNP levels upon ED presentation and outcomes, receiver operator characteristics (ROC) analysis and univariate and multivariate logistic regression analysis were performed, after adjusting for variables which were found to be statistically significant in univariate analysis. A *p* value of <0.05 was considered to be statistically significant.

## 3. Results

### 3.1. Patient Characteristics

Between May 2022 and April 2024, 148 consecutive patients with first-diagnosed or paroxysmal AF were prospectively enrolled upon presentation to the ED. Their mean age was 66 ± 12 years, 47% were females, 44% had first-diagnosed and 56% paroxysmal AF. Their median LVEF fell within the spectrum of preserved ejection fraction, while their median LA diameter was suggestive of mild LA dilatation (Table 1). Median NT-proBNP was 541 pg/mL and eGFR was 83 mL/min/1.73 m^2^. In the majority of patients (85%), cardioversion was performed immediately upon presentation to the ED, while 15% underwent elective cardioversion. EC was performed in 72% of the patients, with PC in the remaining 28% (Table 1). After cardioversion, 43% of patients received oral antiarrhythmics, 83% beta-blockers and 96% anticoagulants. The majority (93%) were discharged directly from the ED, while 7% were admitted to the hospital.

### 3.2. Outcomes

Successful cardioversion to SR was achieved in 121 (82%) out of 148 patients. Compared to patients in whom cardioversion failed to restore SR, patients who were successfully cardioverted did not differ in terms of demographics, type of AF (first-diagnosed or paroxysmal), method of conversion (EC or PC), routine laboratory testing, LVEF, post-cardioversion chronic AF therapies and hospital admission rate (Table 1). However, patients with successful cardioversion were more frequently cardioverted immediately than electively, compared to patients with failed cardioversion (*p* = 0.003), and had lower levels of NT-proBNP [median (IQR), 387 (127–1095) pg/mL vs. 1262 (595–2295) pg/mL, respectively, *p* = 0.004] and a smaller LA diameter [median (IQR), 38 (35–43) mm vs. 44 (38–45) mm, respectively, *p* < 0.001] (Table 1). In addition, NT-proBNP values were higher in patients who underwent elective versus immediate cardioversion [elective vs. immediate: median (IQR), 1314 (507–2093) pg/mL vs. 403 (127–1209) pg/mL, *p* = 0.013].

At D8 post-cardioversion, 124 (84%) out of 148 patients were in SR. Among the 121 patients who had been successfully cardioverted, SR was maintained in 119 (98.3%) patients, while AF recurrence was observed in 2 (1.7%) patients. Of the 27 patients in whom AF cardioversion was unsuccessful, 5 achieved conversion to SR (Table 2). NT-proBNP levels were lower in patients who remained in SR compared to patients with AF recurrence or persistence on D8 [SR vs. non-SR, 370 (127–1095) vs. 1366 (718–2295), *p* = 0.002].

At D30, 127 (86%) out of 148 patients were in SR. AF recurrence was observed in 4 out of the 121 successfully cardioverted patients, while conversion to SR was noted in 10 out of the 27 patients in whom cardioversion was initially unsuccessful (Table 2).

In univariate logistic regression analysis, logNT-proBNP was significantly associated with failure of AF cardioversion at baseline [OR, 95% CI: 4.80 (1.58–14.55), *p* = 0.006] and with AF recurrence or persistence at D8 [OR, 95% CI: 5.61 (1.76–17.89), *p* = 0.004] but not with AF recurrence or persistence at D30 (*p* = 0.088). Additionally, LA diameter was found to be significantly associated with failure of AF cardioversion at baseline [OR, 95% CI: 1.14 (1.05–1.24), *p* = 0.002], as well as with AF recurrence or persistence both at D8 and D30 [D8: OR, 95% CI: 1.13 (1.03–1.23), *p* = 0.007; D30: OR, 95% CI: 1.12 (1.03–1.23), *p* = 0.01]. In multivariate analysis, logNT-proBNP was independently associated with failure of AF cardioversion at baseline [OR, 95% CI: 4.80 (1.58–14.55), *p* = 0.006], as well as with AF recurrence/persistence at D8 [OR, 95% CI: 3.65 (1.06–12.59), *p* = 0.041], after adjusting for LA diameter. Yet, at D30, logNT-proBNP was not able to predict AF recurrence/persistence (*p* > 0.05), which could instead be predicted by LA diameter [OR, 95% CI: 1.17 (1.04–1.31), *p* = 0.007].

**Figure 1:**
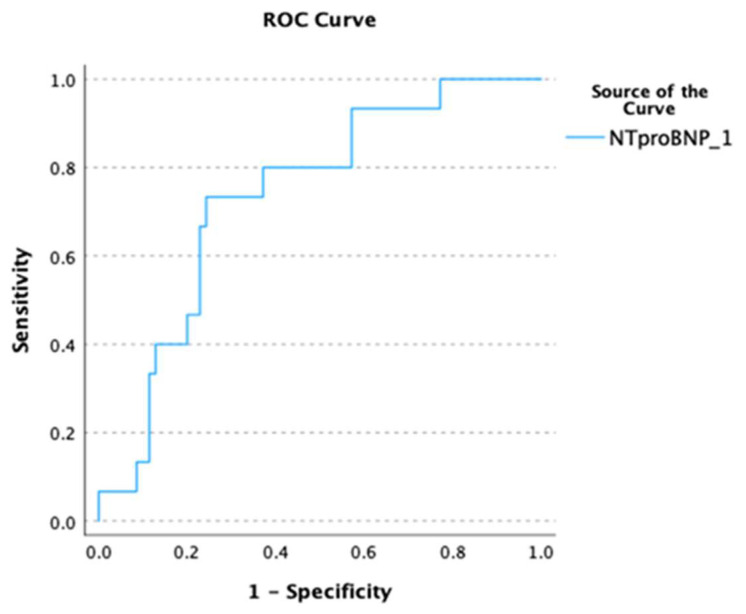
Receiver operating characteristic (ROC) curve depicting the predictive ability of NT-proBNP for failure of AF cardioversion at baseline.

**Figure 2:**
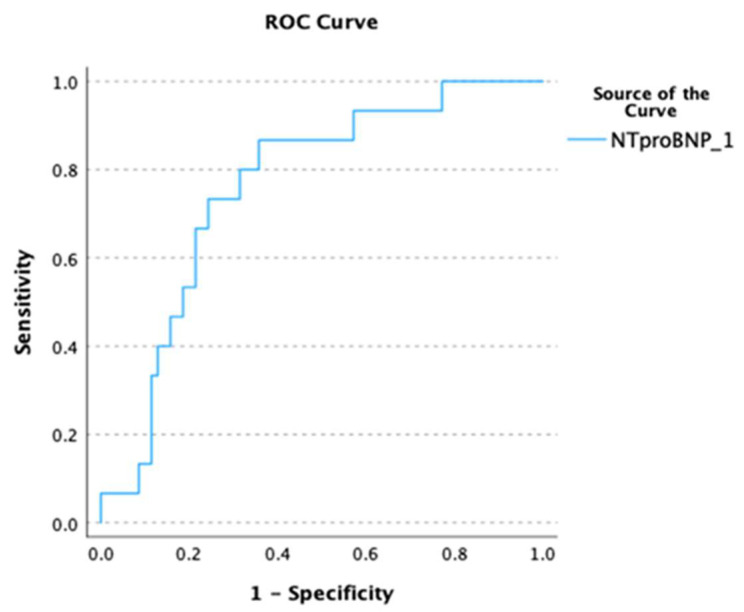
Receiver operating characteristic (ROC) curve depicting the ability of NT-proBNP to predict AF recurrence or persistence at 8-day follow-up after AF cardioversion.

In ROC analysis, NT-proBNP showed good predictive ability in terms of predicting both the outcome of AF cardioversion at baseline and the risk of AF recurrence/persistence at D8 (D0: AUC 0.735, *p* < 0.001, Figure 1; D8: AUC 0.761, *p* < 0.001, Figure 2). A cut-off value of NT-proBNP >580 pg/mL was able to predict failure of AF conversion with 80% sensitivity and 63% specificity as well as the occurrence of recurrent/persistent AF at D8 with 87% sensitivity and 64% specificity.

**Figure 3:**
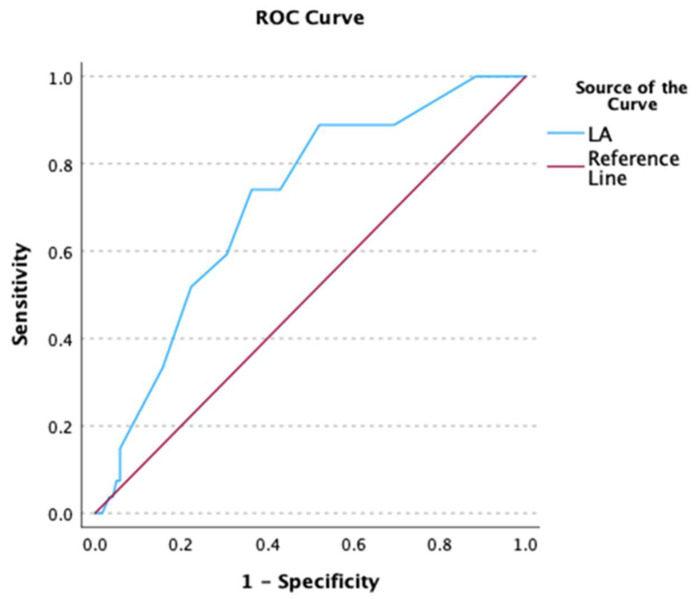
Receiver operating characteristic (ROC) curve of LA diameter as predictor of baseline failure of AF cardioversion.

**Figure 4:**
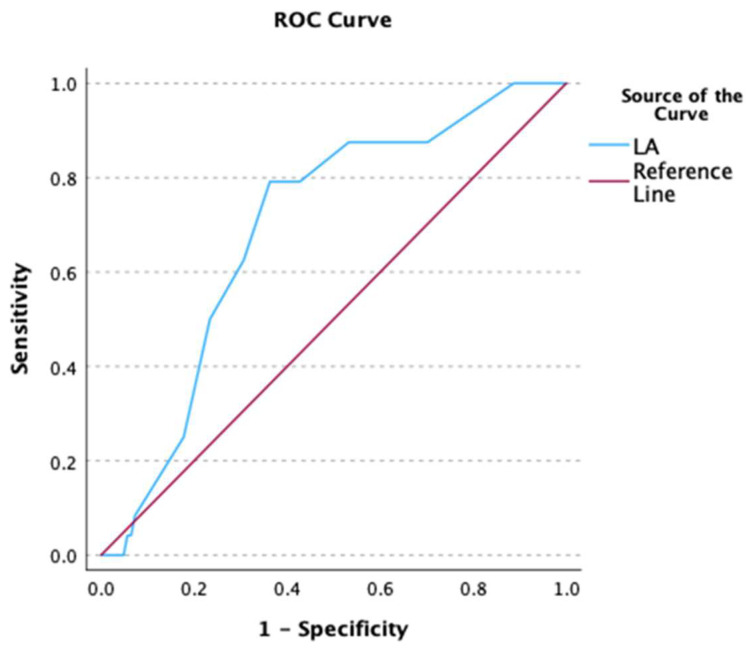
Receiver operating characteristic (ROC) curve of LA diameter as predictor of AF recurrence/persistence at 8 days after cardioversion.

**Figure 5:**
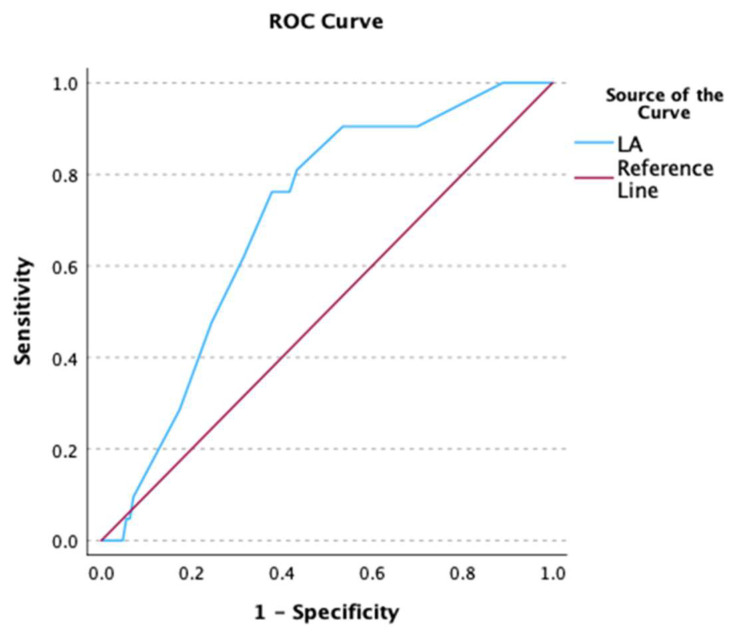
Receiver operating characteristic (ROC) curve of LA diameter as predictor of AF recurrence/persistence at 30 days after cardioversion.

Using ROC analyses, the predictive ability of LA diameter for failure of AF cardioversion at baseline and AF recurrence/persistence at 8 and 30 days after cardioversion was moderate (baseline: AUC 0.711, *p* < 0.001, Figure 3; Day 8: AUC 0.694, *p* < 0.001 Figure 4; Day 30: AUC 0.700, *p* < 0.001, Figure 5).

## 4. Discussion

The present study has shown that, in hemodynamically stable patients presenting to the ED with a first-diagnosed or paroxysmal AF managed with immediate or elective cardioversion, high pre-conversion NT-proBNP levels were associated with increased rates of unsuccessful cardioversion and could predict AF recurrence or persistence early after ED management. Moreover, the predictive value of NT-proBNP was independent of LA diameter, which may suggest that neurohormonal alterations due to AF might be a more significant determinant effective control of the arrhythmia in the short-term than AF-induced atrial remodeling which develops more gradually.

Extensive research has shown an association between natriuretic peptides and AF, regardless of left ventricular hypertrophy or dysfunction. Although the exact pathophysiological mechanisms involved in NT-proBNP release in AF are less clear, it has been suggested that its secretion from the atrial myocytes could be induced by gene upregulation [33] and atrial remodeling due to electrical changes (high atrial rates, shortening of atrial refractory period, increased conduction velocity and ionic alterations) and hemodynamic stressors (atrial overload, stretch and fibrosis) [34,35]. The clinical utility of NT-proBNP in AF extends from predicting the incidence of AF in the general population [36,37,38] with a recommended cut-off value of 124–125 ng/L for at-risk healthy individuals [39,40,41], to predicting AF morbidity and mortality [19,42] and disease progression [20]. Furthermore, natriuretic peptides may show short-term fluctuations in patients with paroxysmal AF [43,44] (increasing in AF recurrence [45], and decreasing after rapid restoration of SR) [46,47,48,49,50,51,52] reflecting AF derangement.

Our findings are in line with previous studies that have investigated the predictive role of baseline NT-proBNP for AF recurrence after cardioversion [45,53,54,55]. A meta-analysis, which included 19 studies with 1373 patients in total, showed that patients with persistent, asymptomatic AF who had undergone elective electrical cardioversion were less likely to maintain SR if their baseline BNP/NT-proBNP levels were elevated [56]. Our study extends these findings by pointing out the role of NT-proBNP in predicting AF recurrence in patients with first-diagnosed or paroxysmal AF who were managed by either EC or PC in the ED. The main difference was that we enrolled patients with recent-onset symptomatic AF who were subjected to immediate or early elective cardioversion in the ED, whereas the aforementioned meta-analysis included patients with persistent, asymptomatic AF who had undergone elective cardioversion. We have also shown an association between NT-proBNP and success of AF cardioversion in these patients. However, previous studies in patients with persistent AF who underwent elective cardioversion have not consistently shown an association between NT-proBNP and the outcome of cardioversion [57,58,59,60]. On the other hand, similar to our study, a previous one in patients with recent-onset AF managed with PC in the ED also showed that low NT-proBNP levels (<450 pg/mL) were associated with a four-fold probability of successful cardioversion, whereas high NT-proBNP (>1800 pg/mL) doubled the probability of unsuccessful cardioversion [61]. Our study confirms the association between NT-proBNP and the success of cardioversion at the ED, which was observed irrespective of the type of cardioversion strategy (PC or EC). Based on the ROC analysis, we propose an NT-proBNP value of <580 pg/mL as a cut-off value to predict both the success of cardioversion and the probability of maintaining SR in the short term. Although rate and rhythm control strategies may not clearly differ in terms of morbidity and mortality [4,62], the presence of debilitating symptoms such as palpitations, reduced exercise capacity, poor quality of life and prevention of long-term complications, may warrant a rhythm control strategy [3,63,64]. Evaluation of NT-proBNP in conjunction with other predictors of unsuccessful cardioversion or AF recurrence (such as age, weight, AF duration, persistent AF, previous AF recurrence, heart failure, and LA diameter) [65,66,67], may serve as a valuable non-invasive tool that would assist clinicians in the ED to tailor appropriate treatment. Recurrence of AF at one year after cardioversion ranges from 65% to 84% [68,69], which can be decreased to 50% with the use of antiarrhythmic medications [69,70]. Therefore, identification of patients who are at greater risk of AF recurrence through NT-proBNP may allow appropriate tailoring of follow-up evaluations (i.e., closer monitoring through additional medical visits, Holter recording and detailed counselling), administration of prophylactic antiarrhythmic drug therapy [3] and strict control of risk factors and comorbidities [71]. Furthermore, patients may benefit from the administration of other pharmacological agents, such as sodium-glucose cotransporter-2 (SGLT-2) inhibitors [72] and glucagon-like peptide 1 (GLP-1) receptor agonists [73] in order to maintain SR after cardioversion.

Our study presents some strengths and limitations. An important point of our study is the enrollment of unselected patients presenting to the ED and excluding patients with structural heart disease and reduced LVEF, which may limit the generalizability of our results [3]. Hence, these circumstances correspond to an actual clinical setting and may enable the development of an appropriate therapeutic plan when encountering patients with paroxysmal or first-diagnosed AF in the emergency setting. One limitation is the short-term follow-up period for the detection of AF recurrence. However, short-term outcomes may reflect more accurately the efficacy of patient management strategies implemented at the ED, whereas long-term outcomes could be influenced to a greater extent by factors post-ED management. Nevertheless, further research should be encouraged in order to determine the value of biomarkers in predicting AF recurrence or persistence in the long-term. This could be achieved by the establishment of multidisciplinary teams, encompassing the collaboration of emergency physicians, cardiologists, family doctors and laboratory healthcare professionals, warranting the monitoring of patients with AF for longer follow-up periods. Another limitation was that the LVEF was assessed via eye-ball method; however, the LVEF was estimated by an experienced and trained emergency physician and was in agreement with the cardiologist’s assessment.

## 5. Conclusions

Pre-conversion NT-proBNP levels are useful in predicting both the cardioversion outcome and the short-term risk of AF recurrence or persistence in patients presenting to the ED with paroxysmal or first-diagnosed symptomatic AF and normal cardiac function. Their evaluation and incorporation into clinical practice may contribute to the implementation of an individualized therapeutic plan when encountering patients with AF in the emergency department.

## Figures and Tables

**Table 1:** Baseline characteristics of study patients.

	All Patients (n = 148)	Successful Cardioversion (n = 121)	Failed Cardioversion (n = 27)	*p*-Value
Age, *years*	66 ± 12	67 (56–75)	69 (62–75)	0.430
Female sex, *n (%)*	70 (47)	58 (48)	12 (44)	0.743
***AF type***				0.358
First-diagnosed, *n (%)*	65 (44)	51 (42)	14 (52)	
Paroxysmal, *n (%)*	83 (56)	70 (58)	13 (48)	
***Method of AF cardioversion***				0.084
Pharmacological, *n (%)*	42 (28)	38 (31)	4 (15)	
Electrical, *n (%)*	106 (72)	83 (69)	23 (85)	
***Timing of AF cardioversion***				**0.003**
Immediate, *n (%)*	126 (85)	108 (89)	18 (67)	
Elective, *n (%)*	22 (15)	13 (11)	9 (33)	
***Laboratory analysis***				
Urea, mg/dL	40 (32–46)	39 (32–46)	42 (27–48)	0.935
Creatinine, mg/dL	0.9 (0.8–1.1)	0.9 (0.8–1.0)	1.0 (0.8–1.1)	0.258
eGFR, mL/min/1.73 m^2^	83 (68–96)	83 (70–97)	83 (54–95)	0.550
TSH, mU/L	1.7 (1.04–2.83)	1.71 (1.05–2.97)	1.61 (0.98–2.46)	0.407
CRP, mg/dL	3.21 (3.18–5.99)	3.21 (3.18–6.36)	3.21 (3.12–5.31)	0.635
NT-proBNP, pg/mL	541 (149–1349)	387 (127–1095)	1262 (595–2295)	**0.004**
Hs troponin T, ng/L	12.2 (6.9–19.3)	12.2 (6.7–19.5)	12.1 (8.58–18.6)	0.842
***Focused echocardiography***				
Left ventricular ejection fraction (LVEF), %	55 (50–60)	55 (50–60)	55 (50–55)	0.066
Left atrial (LA) diameter, mm	38 (35–44)	38 (35–43)	44 (38–45)	**<0.001**
***AF therapy post-cardioversion***				
Antiarrhythmics, *n (%)*	63 (43)	53 (44)	10 (37)	0.520
Beta-blockers, *n (%)*	123 (83)	98 (81)	25 (93)	0.146
Anticoagulants, *n (%)*	142 (96)	115 (95)	27 (100)	0.237
***Hospital admission, n (%)***	10 (7)	6 (5)	4 (15)	0.065

**Table 2:** Patient status (sinus rhythm or atrial fibrillation) at 8-day and 30-day follow-up.

Follow-Up	All Patients (n = 148)	Patients with Initially Successful Cardioversion (n = 121)	Patients with Initially Failed Cardioversion (n = 27)
**8 days post-cardioversion**			
Sinus rhythm (SR), *n (%)*	124 (84%)	119 (98.3%)	5 (18.5%)
Atrial fibrillation (AF), *n (%)*	24 (16%)	2 (1.7%)	22 (81.5%)
**30 days post-cardioversion**			
Sinus rhythm (SR), *n (%)*	127 (86%)	117 (97.7%)	10 (37%)
Atrial fibrillation (AF), *n (%)*	21 (14%)	4 (3.3%)	17 (63%)

## Data Availability

Data are contained within article.
